# Benchmarking post-GWAS analysis tools in major depression: Challenges and implications

**DOI:** 10.3389/fgene.2022.1006903

**Published:** 2022-10-05

**Authors:** Judith Pérez-Granado, Janet Piñero, Laura I. Furlong

**Affiliations:** ^1^ Research Programme on Biomedical Informatics (GRIB), Hospital Del Mar Medical Research Institute (IMIM), Department of Medicine and Life Sciences (MELIS), Universitat Pompeu Fabra (UPF), Barcelona, Spain; ^2^ MedBioinformatics Solutions SL, Barcelona, Spain

**Keywords:** fine-mapping, colocalization, post-GWAS, major depression, eQTLs

## Abstract

Our knowledge of complex disorders has increased in the last years thanks to the identification of genetic variants (GVs) significantly associated with disease phenotypes by genome-wide association studies (GWAS). However, we do not understand yet how these GVs functionally impact disease pathogenesis or their underlying biological mechanisms. Among the multiple post-GWAS methods available, fine-mapping and colocalization approaches are commonly used to identify causal GVs, meaning those with a biological effect on the trait, and their functional effects. Despite the variety of post-GWAS tools available, there is no guideline for method eligibility or validity, even though these methods work under different assumptions when accounting for linkage disequilibrium and integrating molecular annotation data. Moreover, there is no benchmarking of the available tools. In this context, we have applied two different fine-mapping and colocalization methods to the same GWAS on major depression (MD) and expression quantitative trait loci (eQTL) datasets. Our goal is to perform a systematic comparison of the results obtained by the different tools. To that end, we have evaluated their results at different levels: fine-mapped and colocalizing GVs, their target genes and tissue specificity according to gene expression information, as well as the biological processes in which they are involved. Our findings highlight the importance of fine-mapping as a key step for subsequent analysis. Notably, the colocalizing variants, altered genes and targeted tissues differed between methods, even regarding their biological implications. This contribution illustrates an important issue in post-GWAS analysis with relevant consequences on the use of GWAS results for elucidation of disease pathobiology, drug target prioritization and biomarker discovery.

## Introduction

More than 207,400 genetic variants (GVs) have been associated with complex diseases since the introduction of genome-wide association studies (GWAS) (Dehghan, 2018; Buniello et al., 2019). The vast majority of identified GVs lie in non-coding regions of the genome with no clear impact on gene function and disease pathogenesis (Brandes et al., 2022), posing challenges in interpreting the association of the GV with the disease phenotype. Furthermore, these GVs may not be the causal ones but may be in linkage disequilibrium (LD) with the true causal GVs (Visscher et al., 2017; Brandes et al., 2022). We refer to causal GVs to those with a biological impact. A variety of approaches are available to unravel the functional role of GVs identified by GWAS (Kichaev et al., 2014; Amlie-Wolf et al., 2018; Wallace, 2021; Gazal et al., 2022). In addition, there are a plethora of different tools available that serve the same purpose but work with different types of data (e.g., genotype data *versus* full genome summary statistics), under different assumptions (e.g., one causal GV or more), and with diverse outcomes (e.g., causal GVs or relevant gene-cell type combination) (Cano-Gamez and Trynka, 2020; Adebiyi et al., 2021). There is, however, no guideline for determining which tool is best to use for each approach nor a gold standard for evaluating the validity of the results. Furthermore, in contrast to other areas where benchmarking evaluations of methods are in place, such as for protein structure prediction (Protein Structure Prediction Center, 2020) or disease module identification (Dream Challenges, 2022), among others, methods for GWAS data analysis have not been objectively benchmarked. Selecting the right tool is critical in post-GWAS analysis, to properly unravel the functional mechanisms by which the GVs lead to disease, and where different performances can lead to different results (Wen et al., 2017; Rüeger et al., 2018; LaPierre et al., 2021).

**FIGURE 1:**
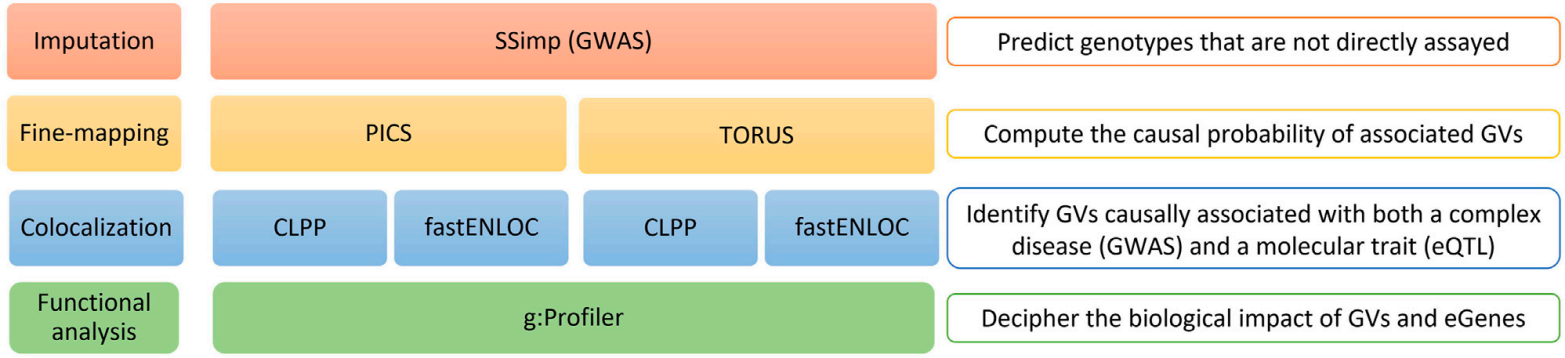
Overview of the study workflow. Schematic representation of the entire analysis workflow: 1) SSimp Imputation; 2) Alternative fine-mapping with PICS and TORUS; 3) Alternative colocalization analysis with CLPP and fastENLOC to both PICS and TORUS fine-mapping results; and 4) Functional analysis of the GVs and eGenes obtained at the end of the workflow. SSimp, Summary Statistics Imputation software; GWAS, genome-wide association studies; PICS, Probabilistic Identification of Causal SNPs; CLPP, Colocalization Posterior Probability; fastENLOC, Fast Enrichment Estimation Aided Colocalization Analysis; GVs, genetic variants; eGenes, genes regulated by expression quantitative trait loci.

There is an absence of a benchmark dataset to assess the performance of post-GWAS analysis tools. Therefore, we propose a systematic and objective comparison of the results obtained by different tools when applied to the same datasets. We designed a fine-mapping and colocalization workflow with different tools running alternatively. Fine-mapping analysis identifies the causal GVs and is a necessary step in most post-GWAS analyses. We used the tools Probabilistic Identification of Causal SNPs (PICS) (Taylor et al., 2021) and TORUS (Wen, 2016) as alternative tools for fine-mapping. Colocalization methods pinpoint the GVs causally associated with a phenotype and a molecular trait of interest, such as expression or methylation. We focused our analysis on expression quantitative trait loci (eQTL), to identify GVs with an effect on the expression of genes, from now on referred to as eGenes. We applied two methods for colocalization analysis: the Colocalization Posterior Probability (CLPP) approach (Hormozdiari et al., 2016) and the Fast Enrichment Estimation Aided Colocalization analysis (fastENLOC) (Pividori et al., 2020; Hukku et al., 2021). We applied the fine-mapping and colocalization workflow to the same GWAS on major depression (MD) and eQTL datasets (Figure 1). The results obtained with each combination of tools were evaluated in terms of fine-mapped and colocalizing GVs, the retrieved eGenes, the tissues in which this regulation of gene expression might take place, as well as the biological processes in which these genes are involved.

The results of the workflow reveal divergence across tools, pinpointing a relevant issue in post-GWAS analysis derived from the lack of method benchmarking. Our findings demonstrate how critical is the fine-mapping step to subsequent analysis and how colocalization outcomes are in turn highly impacted by the assumptions of each tool. As a consequence, the causal GVs and eGenes identified are different and are involved in different biological processes. Overall, given the lack of agreement among tools, we highlight the need for an objective and unbiased assessment of post-GWAS analysis methods and tools to properly leverage GWAS data.

## Materials and methods

Among the plethora of available methods for post-GWAS analysis and which have been reviewed elsewhere (Cano-Gamez and Trynka, 2020; Adebiyi et al., 2021), we focused on fine-mapping and colocalization. Then, we conducted a tool selection based on: workability with full-genome summary statistics, documentation quality, software maturity and developer support availability.

The workflow we describe in this manuscript applies alternative tools for post-GWAS analysis to compare their outcomes (Figure 1). We begin with an imputation step, followed by a fine-mapping and colocalization analysis using two different tools for each of these processes, and finish with a functional analysis of the results obtained using different tools and databases. We present below a more detailed explanation of each step.

### GWAS dataset and imputation

We have selected the latest genome-wide association study (GWAS) on major depression (MD) with publicly available full-genome summary statistics (GCST005902) (Howard et al., 2018). This GWAS evaluated 7,666,894 genetic variants (GVs) in 322,580 European participants (113,769 cases and 208,811 controls). We used the harmonized version of this GWAS dataset. This implies the genomic position is reported against the latest genome build (GRCh38) and the orientation is checked by flipping the effect allele (ie., the allele that confers the risk, which is not always the minor allele) and other alleles whenever appropriate. The beta and 95% confidence interval is also inverted accordingly [downloaded from: http://ftp.ebi.ac.uk/pub/databases/gwas/summary_statistics/GCST005001-GCST006000/GCST005902/harmonised/29662059-GCST005902-EFO_0003761.h.tsv.gz].

The Genotype-Tissue Expression (GTEx) expression quantitative trait loci (eQTL) dataset contains single-tissue cis-eQTL data with eGene, meaning genes regulated by eQTL, and significant variant-gene associations for 49 tissues [downloaded from: https://www.gtexportal.org/home/datasets] (Genotype-Tissue Expression, 2017).

To match the GTEx eQTL panel, we imputed not genotyped GVs in the MD dataset with the Summary Statistics Imputation software (SSimp) (Rüeger et al., 2018). The parameters we used were GWAS full-genome summary statistics GVs with their matching z-scores, reference and effect alleles along with the European 1,000 genomes linkage disequilibrium (LD) reference panel [downloaded from: http://hgdownload.cse.ucsc.edu/gbdb/hg19/1000Genomes/phase3/]. We computed the z-scores by dividing GVs’ effect size, understood as the effect of the risk allele relative to the reference allele, over the standard error (Shi, 2017). We then assessed the imputation quality returned by the SSimp software using the r2. pred parameter, which ranges between 0 -bad quality- and 1 -good quality-. Note that we only considered single nucleotide polymorphisms (SNPs) for this analysis.

### Fine-mapping with PICS and TORUS

Before applying the Probabilistic Identification of Causal SNPs (PICS) and TORUS fine-mapping tools, we matched GWAS GVs and eQTLs to their corresponding LD blocks using the European 1,000 Genomes LD reference panel (Berisa and Pickrell, 2016).

We run PICS by programmatically accessing its web application form. We used LD-based PICS (https://pics2.ucsf.edu/pics2-LD.html), which performs LD expansion and fine-mapping. In brief, PICS takes the most significant GV per association locus along with its associated p-value, performs LD expansion and then computes the probabilities by performing empirical permutations per GV. For GWAS, we submitted the data and obtained the computed PICS probabilities for the input GVs and those in LD, from now on linked GVs. As for eQTLs, we downloaded precomputed LD-based PICS for all GTEx best eQTLs per gene per tissue type [downloaded from: https://pics2.ucsf.edu/Downloads/GTEx/].

We executed TORUS software package using the parameters “-load_zval -dump_pip”. TORUS accepts full-genome summary statistics data, meaning all GVs analysed in the study, and their associated z-scores. Then it computes the causal probabilities using an expectation-maximization algorithm which assumes there is only one causal GV per locus. We obtained these probabilities for all GWAS GVs and GTEx eQTLs (v8) [downloaded from: https://storage.googleapis.com/gtex_analysis_v8/single_tissue_qtl_data/GTEx_Analysis_v8_eQTL.tar], per tissue.

The major histocompatibility complex region (chr6: 28,510,120–33,480,577, GRCh38) was excluded from all datasets for the analysis due to the complex LD structure of GVs, which may lead to inaccurate results (Ghoussaini et al., 2020).

### Colocalization analysis *via* CLPP and fastENLOC

For the colocalization analysis, we implemented two different approaches in the workflow: the Colocalization Posterior Probability (CLPP) approach and the Fast Enrichment Estimation Aided Colocalization Analysis (fastENLOC). We applied these two methods to the fine-mapping results obtained with both PICS and TORUS to identify the genes regulated by causal GVs, also known as eGenes. Both tools consider that there can be more than one causal GV per association locus. CLPP assumes independence between GWAS and eQTL data while fastENLOC does not and computes the enrichment of GWAS on eQTL data using an embedded function. In addition, fastENLOC not only computes SNP colocalization probabilities (SCP) but also regional colocalization probabilities (RCP) to overcome the inability to narrow down to a single causal SNP, common to all tools. Please note that the tools were run following the guidelines and parameters recommended by the authors. We conducted a CLPP approach by computing the product of PICS probabilities for GWAS and eQTL overlapping linked GVs. Based on previous experience in post-GWAS data analysis, we narrowed down the results to the most likely causal GVs (Farh et al., 2015; Ghoussaini et al., 2020; Pérez-Granado et al., 2022) by filtering GWAS GVs and eQTLs PICS probabilities, as well as their product by >10%. We run fastENLOC with fine-mapped GWAS GVs and eQTLs per tissue using the following parameters: default shrinkage 1) and total variants (7,666,894). We filtered the results by RCP >0.5 and SCP >0.001 (Wen et al., 2017).

### Proximal genes

Common gene mapping practices involve looking at the GVs’ overlapping or nearest downstream and upstream genes, also known as proximal genes or pGenes. We retrieved this genetic information using Ensembl *via* SNPnexus (Oscanoa et al., 2020).

We first identified pGenes associated with GVs from PICS and TORUS fine-mapping results and performed gene-set enrichment analysis on both sets.

Then, for each fine-mapping and colocalization combination of tools, we obtained the pGenes to which the GVs mapped and compared them to the corresponding set of eGenes. We also evaluated each pGenes-eGenes set for their association with disease and performed a gene-set enrichment analysis.

### Functional analysis

For the evaluation of association to disease, we followed two different approaches. When evaluating GVs from fine-mapping results, we used variant association data from DISGENET plus (Piñero et al., 2019; DISGENET plus, 2022). Note that the GWAS under evaluation (Howard et al., 2018) and a meta-analysis that it is a part of (Howard et al., 2019) were removed from DISGENET plus datasets to avoid circularity. As for genes, we used the R package disgenetplus2r (disgenetplus2r, 2022), which contains gene-disease association data, and considered Medical Subject Headings (MeSH) disease classes system for disease grouping.

We performed the gene-set enrichment analysis using g:Profiler *via* the R package gprofiler2 (Raudvere et al., 2019) and the following databases: 1) Gene Ontology (GO) biological processes, molecular functions and cellular processes; 2) Reactome and WikiPathways pathways; 3) miRNA annotations; 4) Human Phenotype Ontology, which focuses on rare Mendelian disorders, and has phenotypic features associated with disease; and 5) DISGENET plus, which has genes’ association data to disease and phenotypic traits (v19). The whole set of known human genes was used as domain scope for the analysis and electronic GO annotations were not considered. Furthermore, to make the functional enrichment analysis more meaningful, we filtered the terms by their specificity using their term size (<1,500 genes), which corresponds to the number of genes associated with that term.

In addition, we applied a guilt-by-association approach to overcome the lack of functional information for some genes and assign the function of better-characterized neighbours in the interactome. Thus, we used molecular interaction data from IntAct (Orchard et al., 2014) clustered with MONET (Tomasoni et al., 2020) to evaluate whether different eGenes retrieved from the workflow could belong to the same cluster and thus affect the same molecular pathway. We performed a gene-set enrichment analysis of the retrieved clusters filtering by an eGene-cluster genes ratio of 1:50.

We evaluated the fine-mapping and colocalization results at different levels: the tissue specificity, colocalizing causal GVs, their target genes (eGenes) and their biological implications. We examined the results individually and then compared them across tools, with classic approaches (pGenes) and with the results reported in the original publication.

## Results

This study evaluates and compares the outcomes of different fine-mapping and colocalization tools (Figure 1). To accomplish this, we have run our analysis using the same genome-wide association study (GWAS) on major depression (MD) and expression quantitative trait loci (eQTL) datasets. In addition, and in line with our goal, we address the results of each analytical step individually before getting into their biological implications. The workflow begins with an imputation phase (SSimp) to predict the genotypes not directly assayed in the original GWAS. Then, a fine-mapping step with Probabilistic Identification of Causal SNPs (PICS) and TORUS to identify the most likely causal genetic variants (GVs), meaning those likely to have a biological effect on the trait, and compute their causal probabilities. Next, a colocalization analysis using the Colocalization Posterior Probability (CLPP) approach and the fast enrichment estimation aided colocalization (fastENLOC) software, to identify the GVs causally associated with both MD and a change in expression of a target gene. Finally, the functional analysis, leveraging a diversity of databases, aims to decipher the impact of the identified GVs and eGenes, meaning genes regulated by eQTLs.

### GWAS dataset imputation

The original genome-wide association study (GWAS) consisted of 7,624,931 harmonised genetic variants (GVs) and after imputation to predict missing Genotype-Tissue expression (GTEx) eQTLs, we obtained 7,947,219 GVs (ie. a total of 554,824 imputed GVs). The estimated imputation quality provided by SSimp was generally good for all chromosomes (r2. pred >0.8) except for chromosome 17.

### Fine-mapping with PICS and TORUS

**FIGURE 2:**
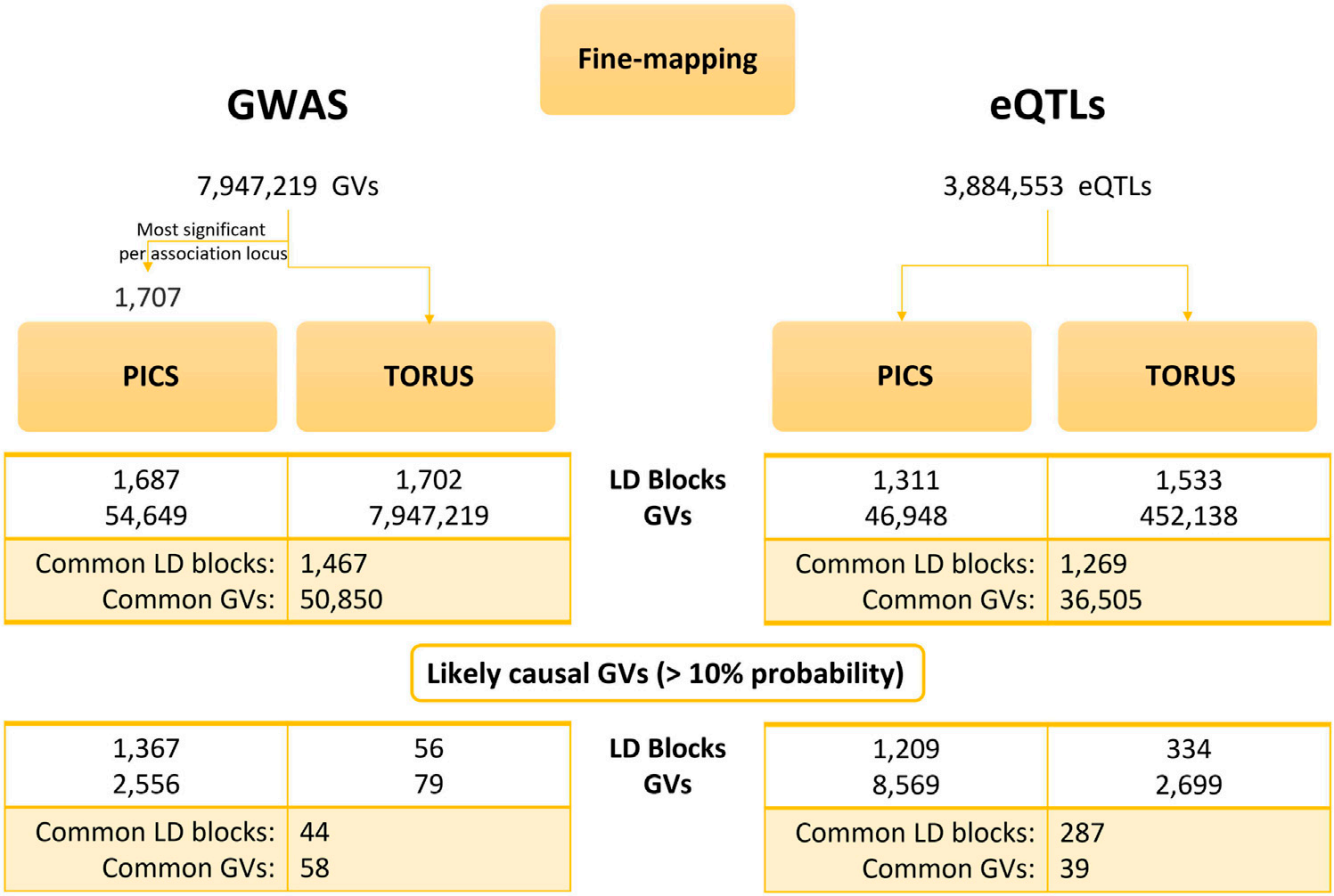
Results of PICS and TORUS fine-mapping analysis. Comparison of PICS and TORUS fine-mapping outcomes at GV and LD block level for both GWAS and eQTL datasets. PICS, Probabilistic Identification of Casual SNPs; GVs, genetic variants; LD, linkage disequilibrium; GWAS, genome-wide association studies; eQTLs, quantitative trait loci.

We run linkage disequilibrium (LD)-based PICS by inputting the most significant GWAS GVs per LD block (1,707 GVs) along with their p-values (Figure 2). After PICS LD expansion and fine-mapping, we obtained 54,649 GVs with their corresponding PICS probabilities. As for the GTEx eQTLs, we downloaded the precomputed LD-based PICS per tissue from the data portal. In parallel, we computed the z-scores for all GWAS GVs and GTEx eQTLs and along with the LD block specification, we used them as input for TORUS.

**FIGURE 3:**
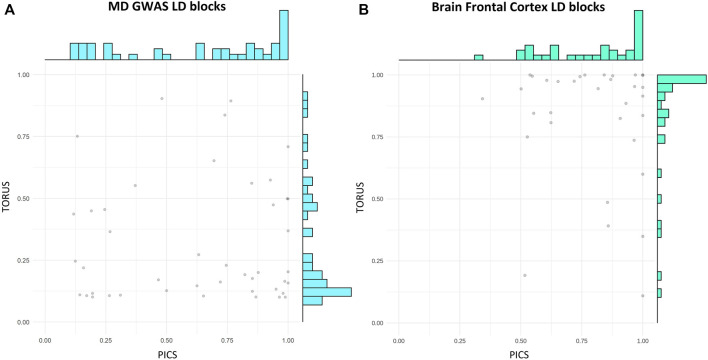
PICS and TORUS fine-mapping probabilities have different distributions. Scatter plot and distribution of PICS and TORUS probabilities for LD blocks containing GVs with PICS and TORUS probabilities >10%. **(A)** MD GWAS and **(B)** Brain Frontal Cortex LD block. PICS, Probabilistic Identification of Causal SNPs; LD, linkage disequilibrium; GVs, genetic variants; MD, major depression; GWAS, genome-wide association studies.

We compared PICS and TORUS initial fine-mapping results (Supplementary Figure S1) and then filtered GVs by a probability >10% to keep the most likely causal GVs. Because each tool has its own assumptions and different GVs could be identified, but these may be in LD, the comparison was done considering the probabilities per LD block. In addition, we examined the distribution of PICS and TORUS sum of probabilities for all LD blocks with likely causal GVs (GWAS: 1,367 and 56, respectively; GTEx: 1,209 and 334, respectively) (Supplementary Figure S1A) as well as the common ones (44 and 287, respectively) (Figure 3A). PICS probabilities for GWAS GVs are biased towards higher values in all cases, with 74% of GWAS LD blocks having a probability greater than 50%. Meanwhile, TORUS probability distribution is skewed towards lower values with only 21% of LD blocks surpassing the 50% probability. Regarding GTEx eQTLs, PICS and TORUS results generally follow a more similar distribution with probabilities biased towards higher values, especially when only GVs with probabilities greater than 10% are considered (Figure 3B and Supplementary Figure S1B; note that we focused on Brain Frontal Cortex region because it is relevant to MD and for illustrative purposes).

The analysis of PICS and TORUS most likely causal GVs (probability >10%) revealed that both sets are enriched in GVs associated with MD, bipolar disorder and other psychiatric disorders (Supplementary Tables S1, S2). PICS causal GVs are also enriched in metabolic-related traits such as triglycerides measurement.

Additionally, we applied classic gene-mapping approaches to PICS and TORUS fine-mapping results, yielding 1,277 and 1,248 proximal genes or pGenes, respectively. Both sets were enriched in genes associated with neurogenesis as well as neuron differentiation and development (Supplementary Tables S3, S4).

### Colocalization analysis *via* CLPP and fastENLOC

The colocalization results from CLPP approach using PICS fine-mapping results yielded 44 GVs and 43 genes regulated by eQTLS, also known as eGenes, affecting 28 tissues (Supplementary Table S5), whereas no results were obtained when using TORUS causal GVs. In parallel, fastENLOC applied to causal GVs identified by PICS resulted in 24 GVs and 17 eGenes across 13 tissues (Supplementary Table S6), while when applied on TORUS probabilities yielded 10 GVs and 3 eGenes in 2 tissues (Supplementary Table S7).

**FIGURE 4:**
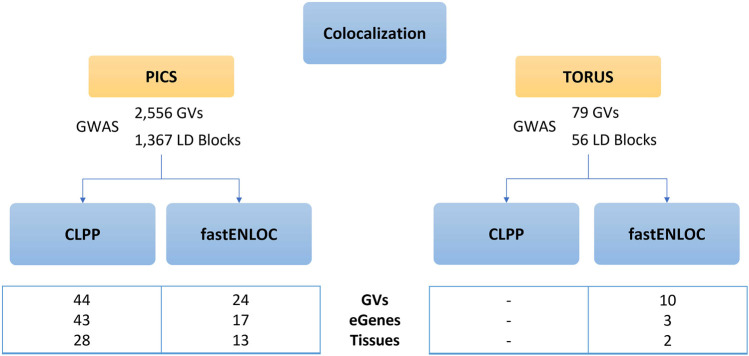
Results of CLPP and fastENLOC colocalization analysis. Comparison of CLPP and fastENLOC colocalization outcomes according to the prior fine-mapping tool used. CLPP, Colocalization Posterior Probability; fastENLOC, Fast Enrichment Estimation Aided Colocalization Analysis; GVs, genetic variants; eGenes, genes regulated by an expression quantitative trait loci.

When comparing methods, the use of different colocalization tools after fine-mapping with PICS yields the most similar results. When using PICS, all tissues and eGenes identified by fastENLOC are also obtained by CLPP, with differences found at the GV level, and CLPP retrieving additional eGenes compared to fastENLOC (Supplementary Table S8 and Figure 4). Meanwhile, when comparing the use of PICS or TORUS fine-mapping probabilities followed by fastENLOC, we only identified one common tissue but with different eGenes and GVs. Similarly, PICS+CLPP and TORUS+fastENLOC yielded common findings only at the tissue level. Among the tissues with causal GVs and eGenes retrieved when using PICS and either colocalization tools, we can find diverse brain regions like the frontal cortex or hypothalamus.

### Proximal genes and functional analysis

We compared eGenes from fine-mapping and colocalization workflow to pGenes from PICS and TORUS fine-mapping results. Only 3 genes overlapped between pGenes from PICS fine-mapping and PICS+CLPP eGenes (KTN1, PXMP4 and ESYT2) and one with PICS+fastENLOC (KTN1). There was no overlap with pGenes when comparing to TORUS. The eGenes from PICS+CLPP are enriched in their association with miRNAs and the eGenes from PICS+fastENLOC in RNA Polymerase I Promoter Escape (Supplementary Table S9). Considering all eGenes together (46), these are functionally enriched in terms related to transcription factor regulation and miRNA. We also assessed the distribution of the eGenes in a clusterized human interactome. The three sets of eGenes (i.e., PICS+CLPP, PICS+fastENLOC and TORUS+fastENLOC) belonged to different clusters, except for eGenes shared across tools results (ie. 17 shared eGenes which are located in 10 clusters). Some of these clusters were associated with transcription factor regulation, inflammation or neurogenesis (Supplementary Tables S10, S11). No clusters identified for TORUS+fastENLOC passed the functional analysis filters, that is a ratio of eGenes over cluster genes higher than 1:50 and enriched term size <1,500 genes.

When we applied traditional gene mapping approaches to the GVs that were found to regulate the expression of those eGenes, we discovered a total of 74 pGenes. The vast majority of eGenes identified do not match pGenes, which holds true across all workflows (Table 1). In addition, most matches derive from GVs lying in an intronic region of the genome (Supplementary Table S12). Nonetheless, all sets of pGenes and eGenes are associated with mental disorders, behaviour and behaviour mechanisms as well as psychological phenomena and processes and nervous system disease (Supplementary Figures S2, S3). Additionally, pGenes are enriched in GO terms associated with diverse signalling pathways (Supplementary Table S13).

**TABLE 1:** Identified eGenes differ from classic gene mapping (pGenes).

	eGenes	pGenes	Matches
PICS + CLPP	43	64	10
PICS + fastENLOC	17	31	3
TORUS + fastENLOC	3	8	0

eGenes, genes regulated by expression quantitative trait loci; pGenes, proximal genes; PICS, Probabilistic Identification of Causal SNPs; CLPP, Colocalization Posterior Probability; fastENLOC, fast enrichment estimation aided colocalization analysis. The number of eGenes, also known as genes regulated by eQTLs, retrieved from the fine-mapping and colocalization analysis; the number of pGenes or proximal genes, that is overlapping or nearest upstream and downstream genes; and matches between eGenes and pGenes. The information is shown for each combination of fine-mapping and colocalization tools used.

Furthermore, we compared the results obtained with the original publication where 14 GVs and 7 pGenes were reported. The latter are functionally associated with synapsis (Supplementary Table S13) and 6 of them have a prior association with mental disorders (Supplementary Figure S4). Only 5 fine-mapped GVs from PICS and 7 GVs from TORUS overlapped with the GVs reported in the original publication, and 1 pGene (SGIP1), which is in both sets of fine-mapped pGenes. However, none of the GVs and pGenes obtained by colocalization with any combination of tools evaluated in our pipeline overlapped with the GVs and pGenes reported in the original publication.

## Discussion

Currently, there are a plethora of strategies available for post-GWAS analysis (Cano-Gamez and Trynka, 2020; Adebiyi et al., 2021). Here, we have focused on two main approaches: fine-mapping, which aims to identify the likely causal GVs, and colocalization, aimed at identifying which genes are regulated by the GVs at the expression level (eGenes). Furthermore, while many tools address the same goal, there is no standard set of causal GVs that have been experimentally validated for benchmarking to determine and compare which one is the most adequate (Brandes et al., 2022). Thus, we have designed an evaluation exercise to assess the outcome of different fine-mapping and colocalization tools using the same MD GWAS and eQTL dataset. To the best of our knowledge, no study goes beyond the comparison of the different tool’s assumptions and thus the evaluation of the biological implications of their findings (Wen et al., 2017; Cano-Gamez and Trynka, 2020).

Our main premise throughout this analysis has been to use each tool as it was intended by following developers’ recommendations and guidelines as closely as possible. This way we could get the most out of them and compare their optimised outcomes. Furthermore, one of the primary reasons behind the tools’ selection was their ability to work with full-genome summary statistics instead of individual genotype data, which can be difficult to obtain due to privacy concerns. Other criteria for tool selection included the quality of documentation, the maturity of the software, and the availability of developer support.

Prior to post-GWAS analysis, the imputation process using SSimp yielded very good quality results except for chromosome 17. One possible explanation is that SSimp provides hg19 1000Genomes phase3 as the reference panel. This version of the genome has some gaps, most of which are found in telomeres and centromeres, having a strong impact on chromosome 17 (Rashid-Kolvear et al., 2007; Genome Reference Consortium, 2022). We then proceeded with the fine-mapping and colocalization workflow, keeping the previously mentioned issue in mind when evaluating their results.

Fine-mapping is significantly influenced by LD patterns and the used tools, PICS and TORUS, which work under different assumptions (see Methods). Therefore, to have comparable results we considered the probabilities obtained at the LD block level, because the most likely causal GVs may differ or not be discernible due to high LD between GVs. In addition, to account for the difference in the number of GVs which could be driving the observed inverse distribution of probabilities between tools (Supplementary Figure S1), only the most likely causal GVs were considered in the comparison (Figure 2). In general, TORUS retrieves GVs with lower probabilities compared to PICS. This could be explained by the algorithm’s conservative nature and its assumption of one causal GV per association locus, with probabilities biased towards zero when the locus contains multiple causal GVs (Wen, 2016). Indeed, the one causal GV assumption has been debated, with multiple GVs acting together resulting in a more reasonable theory (Burgess, 2022). Nonetheless, both PICS and TORUS most likely causal GVs are enriched in their association with MD, bipolar disorder and other psychiatric disorders (Supplementary Tables S1, S2). This suggests that both fine-mapping approaches identify likely causal GVs associated with MD. GVs fine-mapped by PICS are also enriched in diseases and traits usually comorbid with MD such as alcohol consumption (Gémes et al., 2019) and metabolic traits like serum total cholesterol measurement (Gold et al., 2020). Classic gene mapping of PICS and TORUS fine-mapping results (2,556 GVs and 79 GVs respectively, common- 58 GVs) (Figure 2), yielded 1,277 and 1,248 pGenes, respectively, with all TORUS pGenes included in PICS. These results could be explained because, compared to TORUS, PICS computes higher probability values and may retrieve more than one likely causal GV per locus. But provided the set probability threshold, some of these GVs may be in LD and therefore mapping to the same genes. Both sets of pGenes are enriched in genes associated with neurogenesis, highly affected in MD (Li Z. et al., 2021). All in all, fine-mapping is a critical step in post-GWAS analysis, with high divergence observed between different methods, particularly at the level of GVs and their associated probabilities, which will highly impact subsequent colocalization analysis.

CLPP and fastENLOC colocalization approaches were applied to both fine-mapping results from PICS and TORUS. Following the same logic, given that TORUS computed lower probability values, PICS yielded more colocalization findings (Supplementary Tables S5–S7). Furthermore, we have similar results under CLPP assumption of independence between GWAS and eQTLs compared to fastENLOC built-in function to compute their enrichment, with fastENLOC being more stringent as previously described (Hukku et al., 2021). Interestingly, when focusing on a single tissue, the results do not match at the GV level but do so at the eGene level (Supplementary Table S8). This suggests that there might be different GVs that have an effect on the expression of the same eGenes. It also highlights the importance of the identification of eGenes to determine how GVs may ultimately impact the disease phenotype.

The overlap between eGenes and pGenes from PICS and TORUS fine-mapping was very small, with 3 genes in total. Among them, KTN1 has also been associated with MD (Dall’Aglio et al., 2021) and ESYT2 is involved in neurodevelopmental pathways and may be associated with suicidal behaviour trends in MDD although more research is needed (Calabrò et al., 2018). eGenes from PICS+CLPP were functionally enriched with miRNAs. These have been recently reported as relevant in MD pathogenesis and treatment (Dwivedi, 2014). Specifically, hsa-miR-23a-3p has repeatedly been associated with duloxetine treatment response assessment in MD (Kim et al., 2019). Moreover, the eGene GMPPB identified from TORUS+fastENLOC has already been associated with MD pathogenesis in proteome-wide association studies (Wingo et al., 2021). GMPPB is involved in glycosylation, which has been reported as relevant and even hypothesized as a potential biomarker for MD (Yamagata and Nakagawa, 2020). Considering all eGenes together, they are enriched in their association with transcription factor regulation (Supplementary Table S9), which has already been related to MD (Zhong et al., 2019; Li X. et al., 2021; Pérez-Granado et al., 2022). The mapping of eGenes to protein interaction clusters indicated that the three sets of genes (i.e., PICS+CLPP, PICS+fasENLOC and TORUS+fastENLOC) belonged to distinct clusters and are thus likely to be involved in different biological processes. Nevertheless, PICS+CLPP and PICS+fastENLOC associated sets of clusters were enriched with genes associated with processes involving TF regulation as well as inflammation or neurogenesis (Supplementary Tables S10, S11). All these processes are associated with MD pathogenesis (Shadrina et al., 2018; Zhong et al., 2019; Li X. et al., 2021, Li et al., 2021 Z.; Pérez-Granado et al., 2022). In general, the identified eGenes are poorly characterized yet the cluster analysis shades some light on their potential molecular associations.

Fine-mapping and colocalization analysis successfully identified eGenes associated with mental disorders (Supplementary Figures S2–S4) that differed from the set of pGenes, particularly when focusing on non-coding regions of the genome (Table 1 and Supplementary Table S12). Accordingly, pGenes are enriched in their association with pathways that have been reported as disrupted in MD such as MAPK (Wang et al., 2020), ErbB (Ledonne and Mercuri, 2020), PI3K/AKT (Matsuda et al., 2019) and ERK (Wang and Mao, 2019) signalling pathways (Supplementary Table S13); as well as MD potential causes like stress or inflammation (Shadrina et al., 2018; Li Z. et al., 2021). When comparing the results from our workflow to the original manuscript, there were only matches when considering the fine-mapped PICS and TORUS results but not after colocalization analysis. The common pGene between the three datasets was SGIP1, which has been involved in mood regulation (Dvorakova et al., 2021).

Brain regions are of particular interest in MD and as such, we focused the evaluation of our results on them. The brain frontal cortex, hypothalamus, pituitary and brain cerebellar hemisphere have common findings between PICS and both colocalization tools. MD and myclonus-dystonia are usually comorbid, and their association has typically been studied in relation to SGCE mutation and its potential pleiotropic effect (Peall et al., 2013; Kim et al., 2017; Cazurro-Gutiérrez et al., 2021). However, whether SGCE plays a role in MD manifestation has been debated. On the one hand, animal studies have shown that knocking out this gene causes myoclonus, motor coordination deficits, and depression-like behaviour (Cazurro-Gutiérrez et al., 2021) which is consistent with the lower expression levels reported by GTEx. On the other hand, a similar frequency of MD has been reported in SCGE mutated and wild-type myoclonus dystonia patients (Kim et al., 2017). Focusing on the hypothalamus, one of the most common causes of MD is stress, which affects the hypothalamic-pituitary-adrenal axis by increasing glucocorticoid levels (Karger et al., 2018; Oliva et al., 2018). These have an impact on various signalling pathways, including the Wnt pathway, in which FZD5 plays a role, and neurogenesis (Karger et al., 2018). However, the changes in gene expression caused by rs77678807 reported by GTEx are the inverse of what we would expect (Genotype-Tissue Expression, 2017). PCOLCE2 is highly expressed in the pituitary and there is evidence of reduced levels in depression-like behaviours in mice (Yamawaki et al., 2018), consistent with rs9757063 effect. Indeed, it has already been associated with psychiatric disorders by GWAS studies (Martínez-Magaña et al., 2021). However, how exactly they play a role in MD pathogenesis is still unknown. Little is known about the eGenes and GVs identified in the brain cerebellar hemisphere. Additionally, in the brain frontal cortex and hypothalamus, two different lncRNAs have been identified, LINC01159 and RP11-838N2.5 respectively. Even though little is known about them, lncRNAs seem to play a relevant role in MD pathogenesis and therapeutics (Shi et al., 2021; Hao et al., 2022). PICS+CLPP identified rs1480432 as upregulating the expression of DTNA, which is associated with neurogenesis and underregulating the maturation and stability of postsynaptic density (Chen et al., 2022). MAO B has been found to be overexpressed in postmortem brain tissue from MD patients, while DTNA is found to be underexpressed in MAO B knockout mice. The colon is another tissue whose associations with MD have produced intriguing results. ACTL8 is both associated with the microbiome composition and MD, but it is still unclear whether and/or which role the gut microbiome may have in a person’s susceptibility to MD (Martins-Silva et al., 2021).

In general, both classic gene mapping approaches and colocalization analysis identified genes associated with MD or associated relevant processes. Colocalization analysis can provide insights about the effect of GVs located in non-coding regions of the genome, pinpointing the genes they regulate and the relevant tissues. As it has previously been reported the closest gene may not always be the causal one (Brodie et al., 2016; Zhu et al., 2016). These results would need further evaluation with other types of functional genomics data and ultimately experimental validation to verify the role of these regulatory mechanisms in disease pathogenesis (Dehghan, 2018).

Our goal was to illustrate the impact of the lack of standards on the selection of the most adequate post-GWAS analysis method using a fine-mapping and colocalization workflow that compared different tools. The results revealed a high divergence between fine-mapping methods due to their assumptions, which in turn highly impacted the next steps. TORUS one causal variant assumption may tip the balance in favour of PICS considering fine-mapping and posterior analytical steps. Colocalization results seem to diverge in the amount of GVs and eGenes identified, with fastENLOC being more stringent by considering the enrichment of GWAS on eQTLs. All in all, despite the potential of combining GWAS data with molecular profiling datasets to guide in the interpretation of the functional impact of GVs located in non-coding regions of the genome, the results of our analysis revealed shortcomings related with the analytic tools. We propose that objective evaluation and benchmarking of post-GWAS analysis tools is required in order to fully leverage GWAS data for precision medicine and drug R&D applications.

## Data Availability

The original contributions presented in the study are included in the article/Supplementary Material, further inquiries can be directed to the corresponding author.

## Abbreviations

CLPP, Colocalization Posterior Probability; eGenes, genes regulated by eQTLs; eQTL, expression quantitative trait loci; fastENLOC, Fast Enrichment Estimation Aided Colocalization Analysis; GTEx, Genotype-Tissue Expression; GV, genetic variant; GWAS, Genome-Wide Association Studies; LD, linkage disequilibrium; MD, major depression; pGenes, proximal genes; PICS, Probabilistic Identification of Causal SNPs; SNPs, single nucleotide polymorphisms; SSimp, Summary Statistics Imputation software.
