# Supplementary figures and images for: Benchmarking post-GWAS analysis tools in major depression: Challenges and implications
Source: Front Genet. 2022 Oct 5;13:1006903. doi: 10.3389/fgene.2022.1006903 (PMC9579284; doi:10.3389/fgene.2022.1006903)

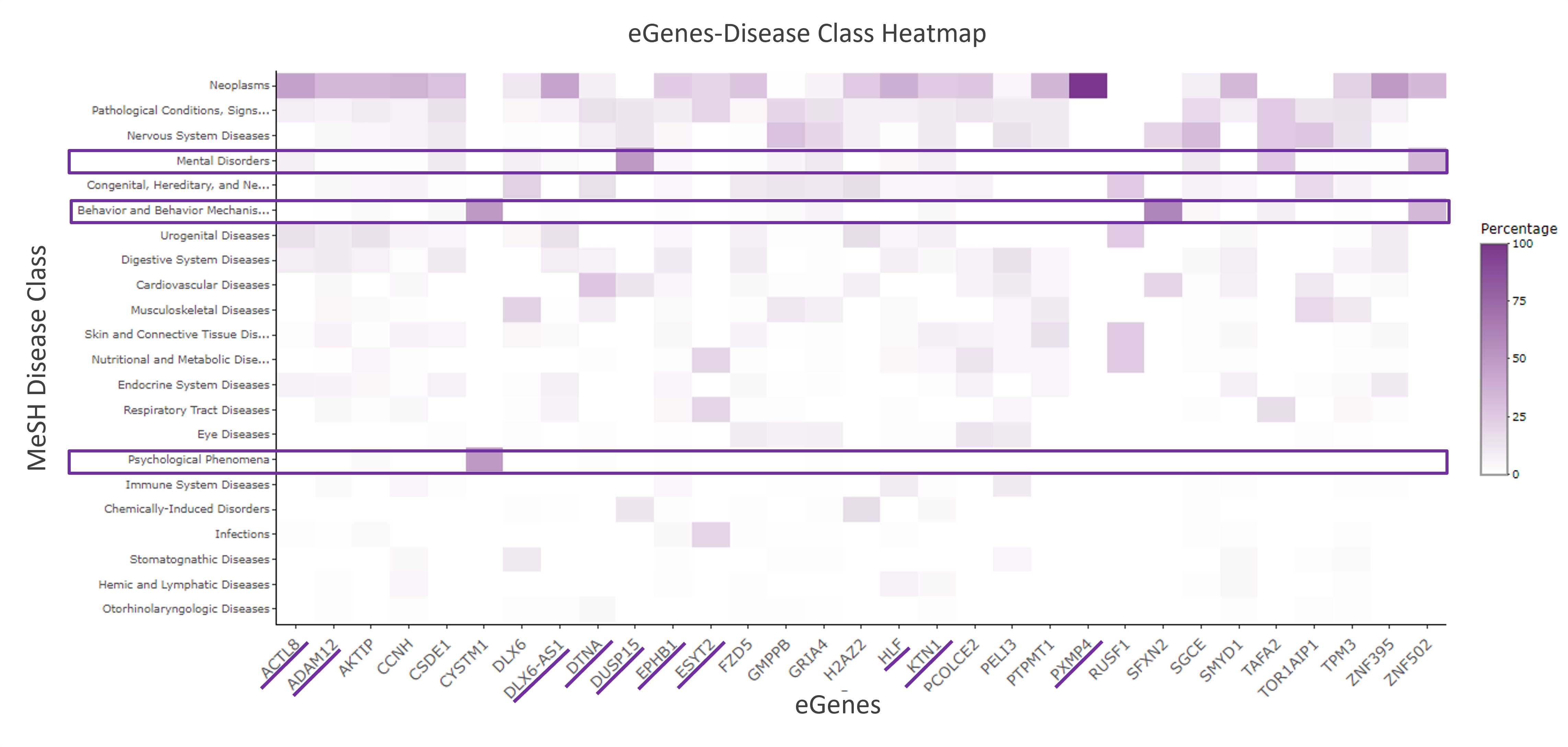

Supplement: Supplementary file 1 [file Image3.JPEG]

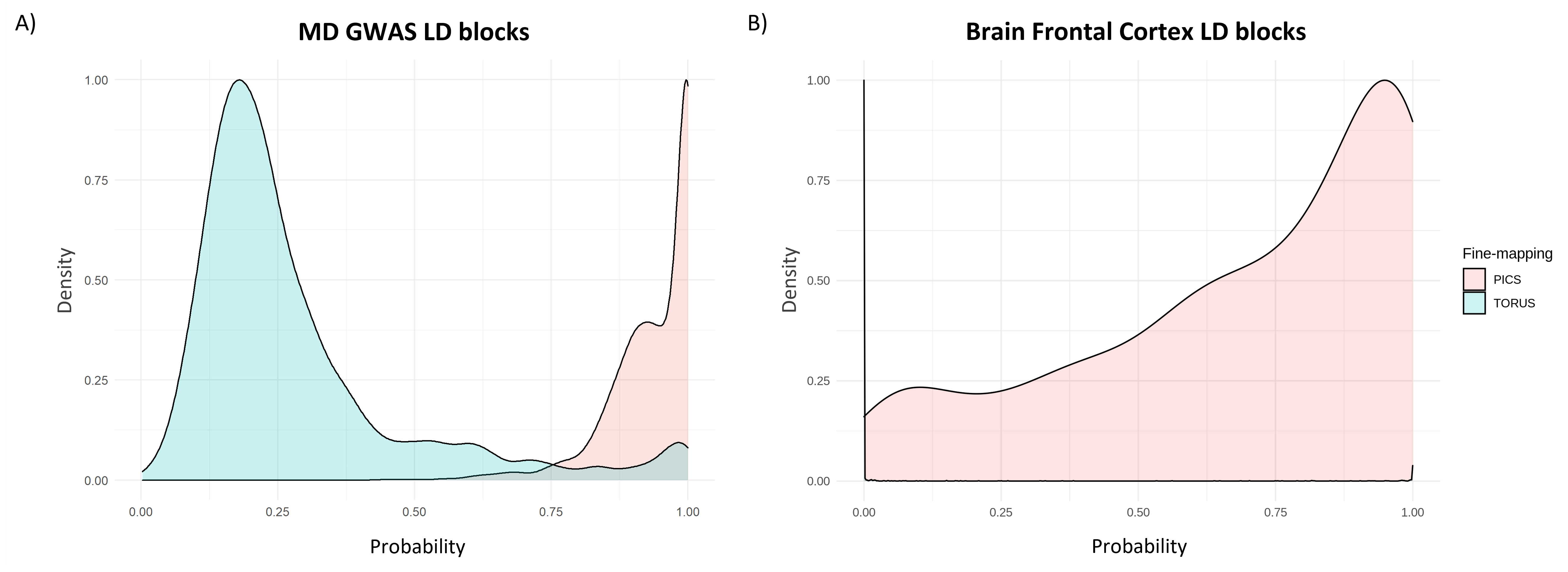

Supplement: Supplementary file 3 [file Image1.JPEG]

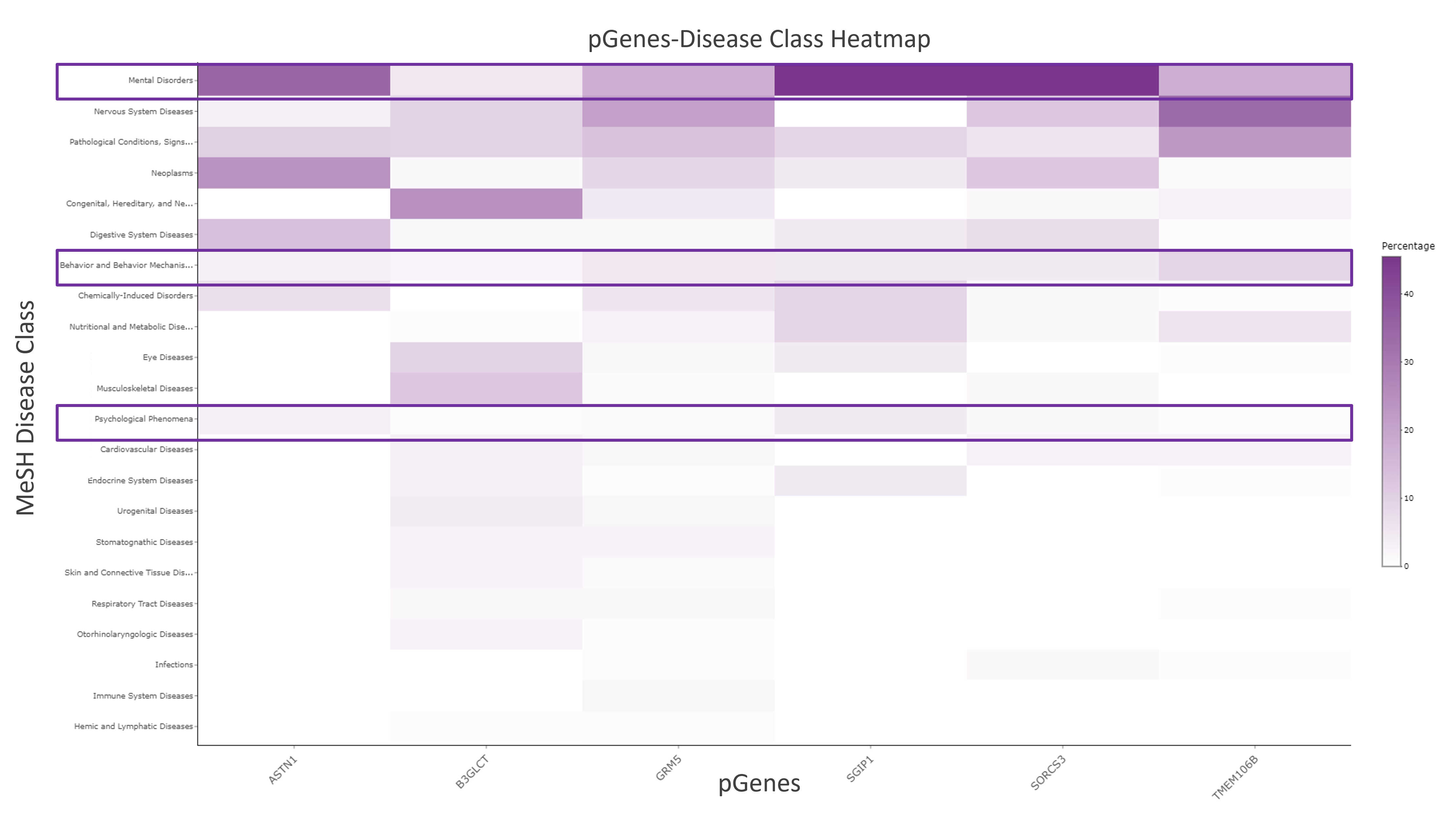

Supplement: Supplementary file 4 [file Image4.JPEG]

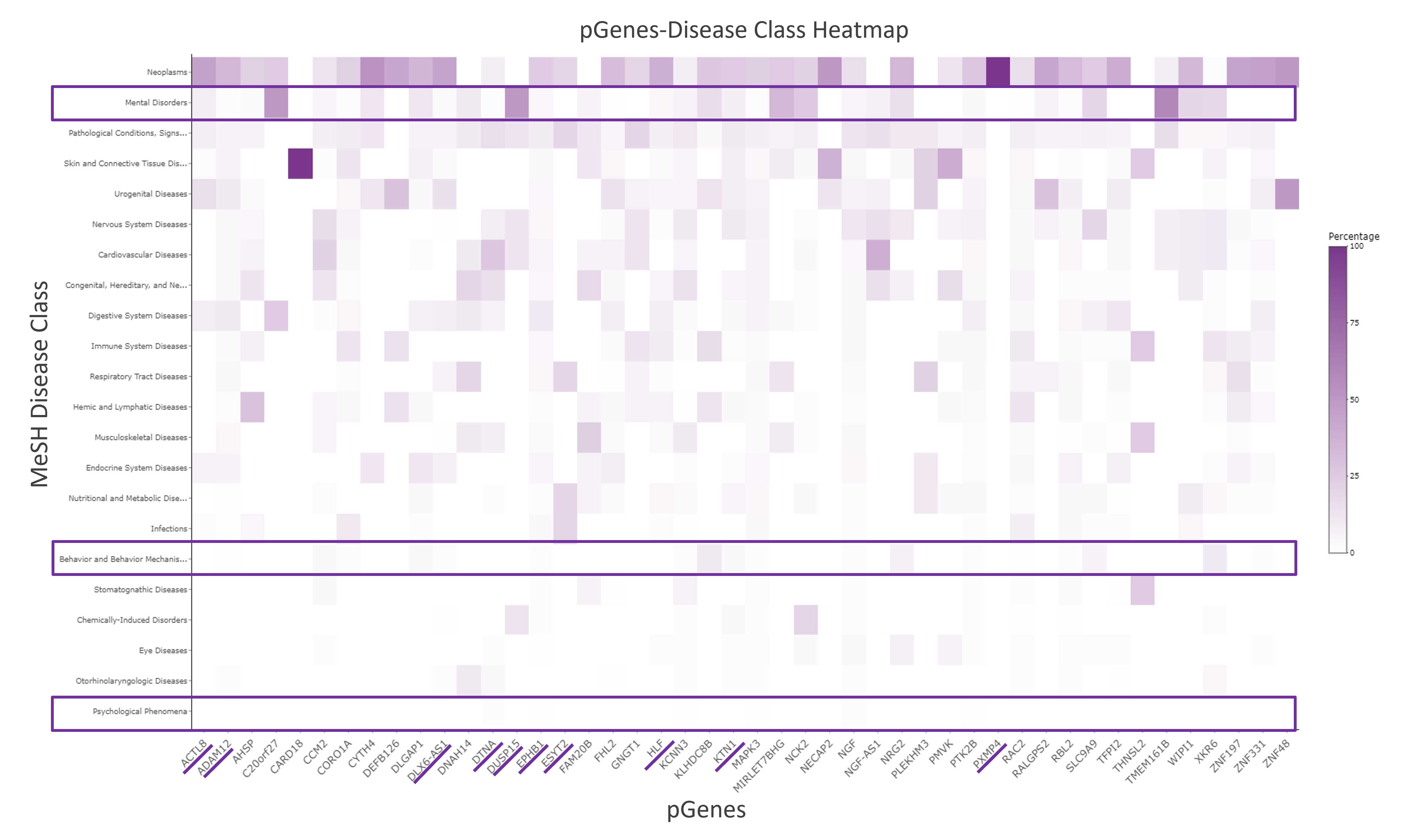

Supplement: Supplementary file 5 [file Image2.JPEG]
