# Supplementary material for: Benchmarking post-GWAS analysis tools in major depression: Challenges and implications
Source: Front Genet. 2022 Oct 5;13:1006903. doi: 10.3389/fgene.2022.1006903 (PMC9579284; doi:10.3389/fgene.2022.1006903)
Supplement: Supplementary file 2 [file Table1.DOCX]

Supplementary Figures

**
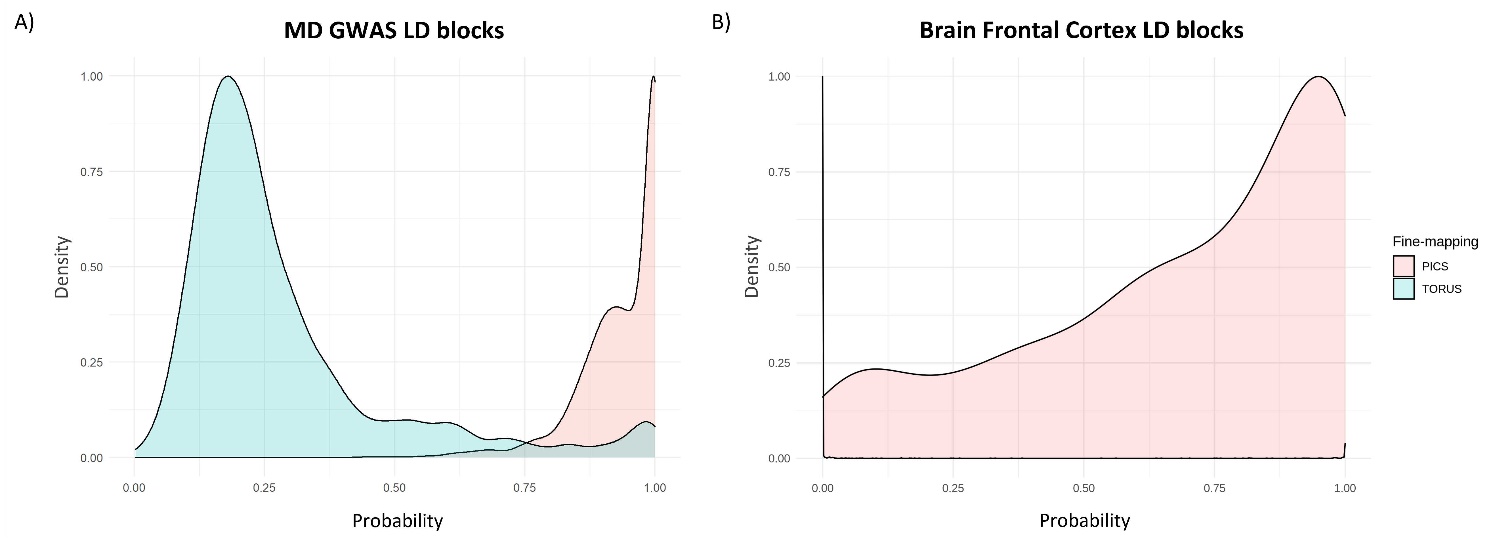
**

**Supplementary Figure S1. Distribution of PICS and TORUS fine-mapping probabilities.** Distribution of LD blocks probabilities for **A)** MD GWAS and **B)** Brain Frontal Cortex eQTLs. PICS: Probabilistic Identification of Causal SNPs; LD: linkage disequilibrium; MD: major depression; GWAS. genome-wide association studies; eQTL: quantitative trait loci.


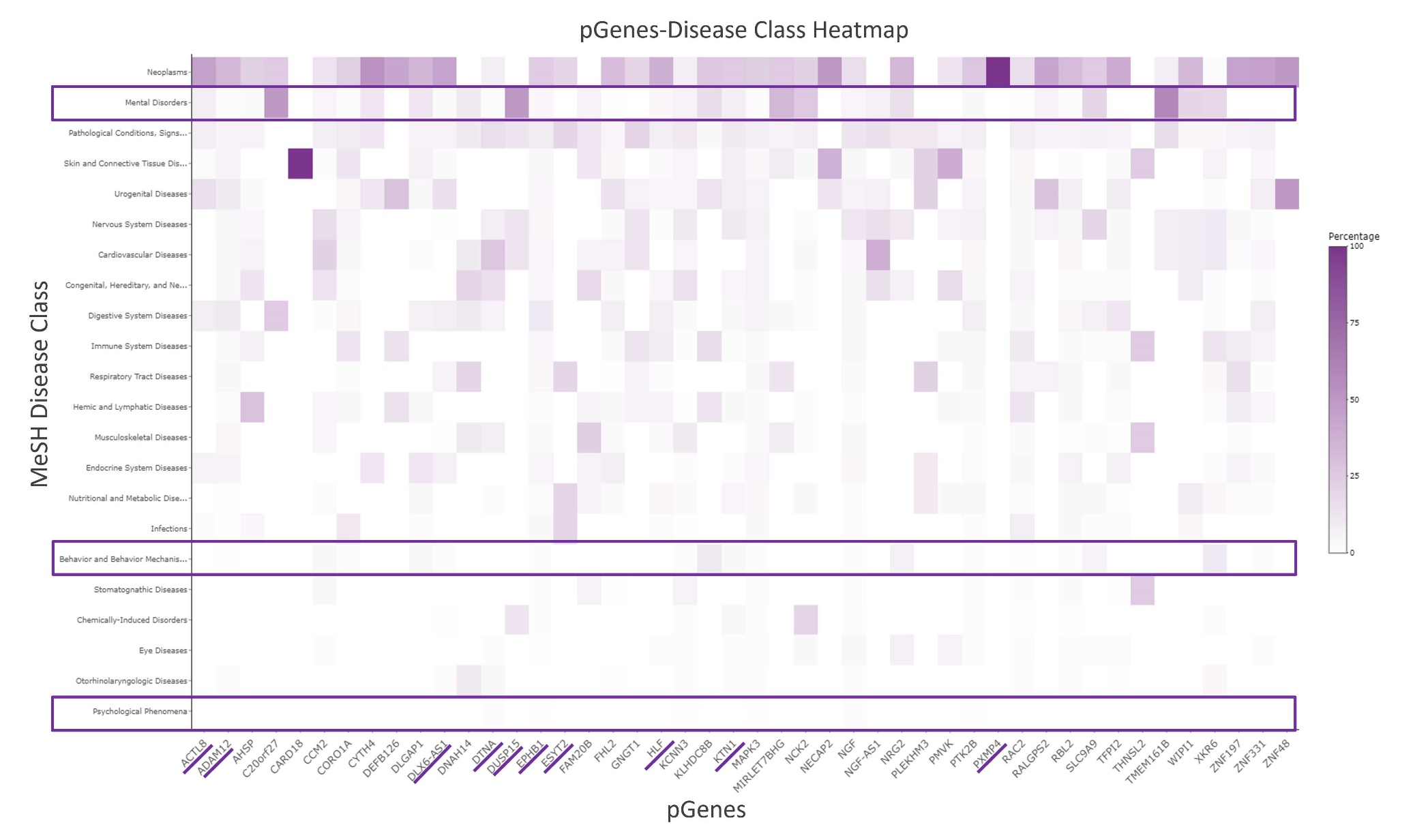


**Supplementary Figure S2. pGenes-Disease class heatmap.** Heatmap with disease classes associated with the list of pGenes. Note that pGenes matching eGenes are underlined and MeSH classes associated with MD are highlighted. The colour scale is proportional to the percentage of diseases in each disease class. pGenes: proximal genes.

**
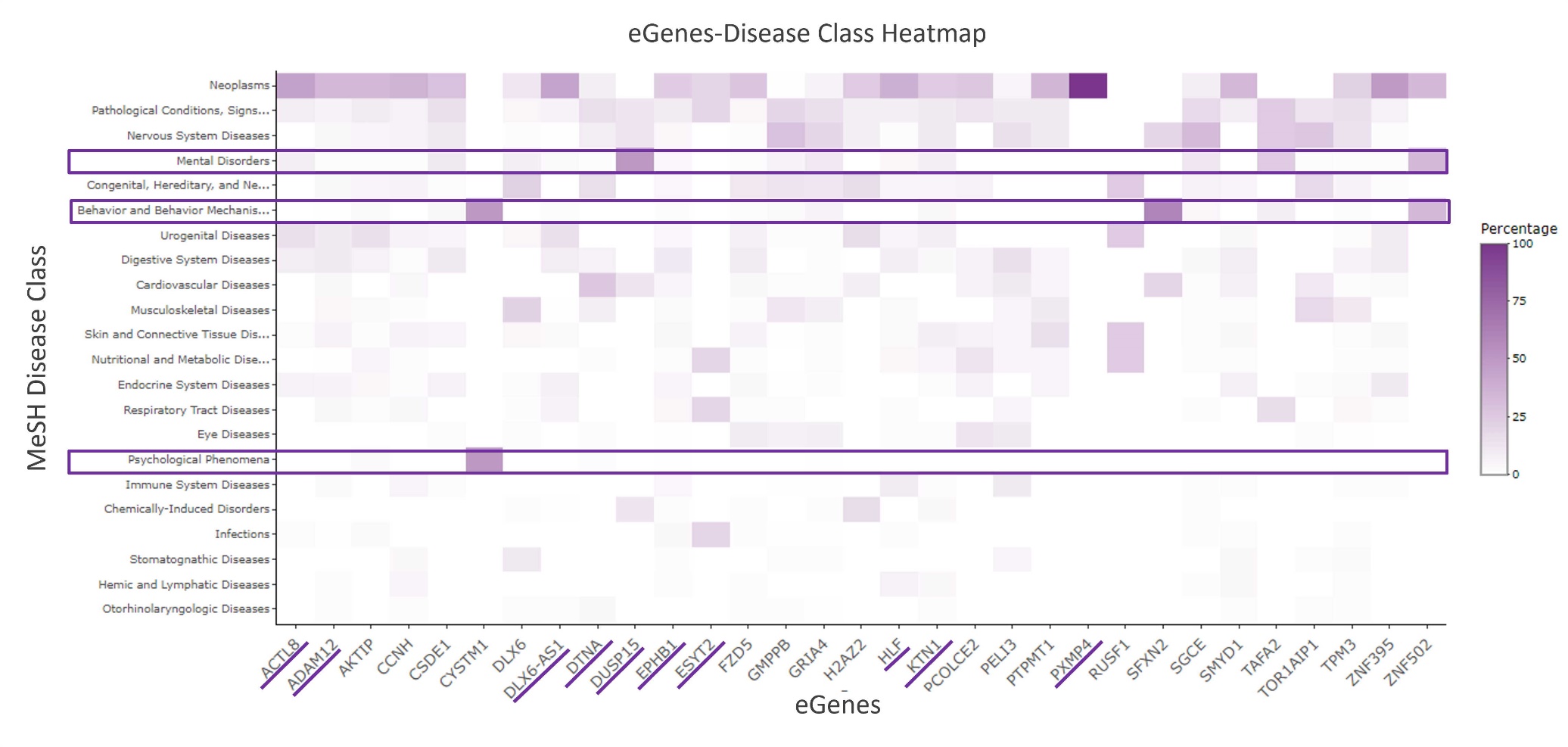
**

**Supplementary Figure S3. eGenes-Disease class heatmap.** Heatmap with disease classes associated with the list of eGenes. Note that eGenes matching pGenes are underlined and MeSH classes associated with MD are highlighted. The colour scale is proportional to the percentage of diseases in each disease class. eGenes: genes regulated by expression quantitative trait loci.

**
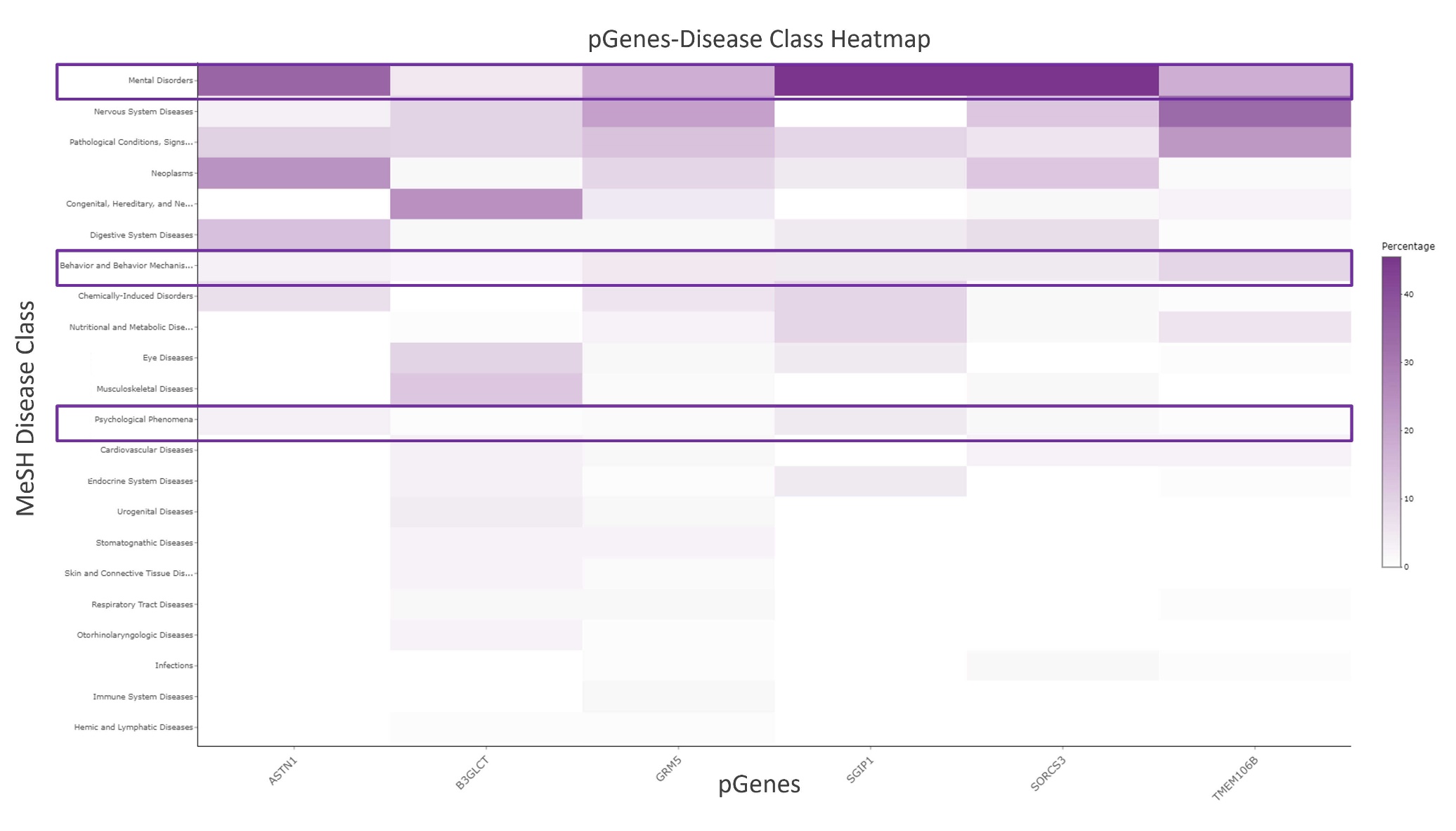
**

**Supplementary Figure S4. Original article pGenes-Disease class heatmap.** Heatmap with disease classes associated with the list of pGenes from the original article. Note that pGenes matching eGenes are underlined and MeSH classes associated with MD are highlighted. The colour scale is proportional to the percentage of diseases in each disease class. pGenes: proximal genes.
